# Supplementary figures and images for: Portability of a text mining algorithm for detecting adverse drug reactions in electronic health records across diverse patient groups in two Dutch hospitals
Source: PLOS Digit Health. 2026 Feb 10;5(2):e0001230. doi: 10.1371/journal.pdig.0001230 (PMC12890107; doi:10.1371/journal.pdig.0001230)

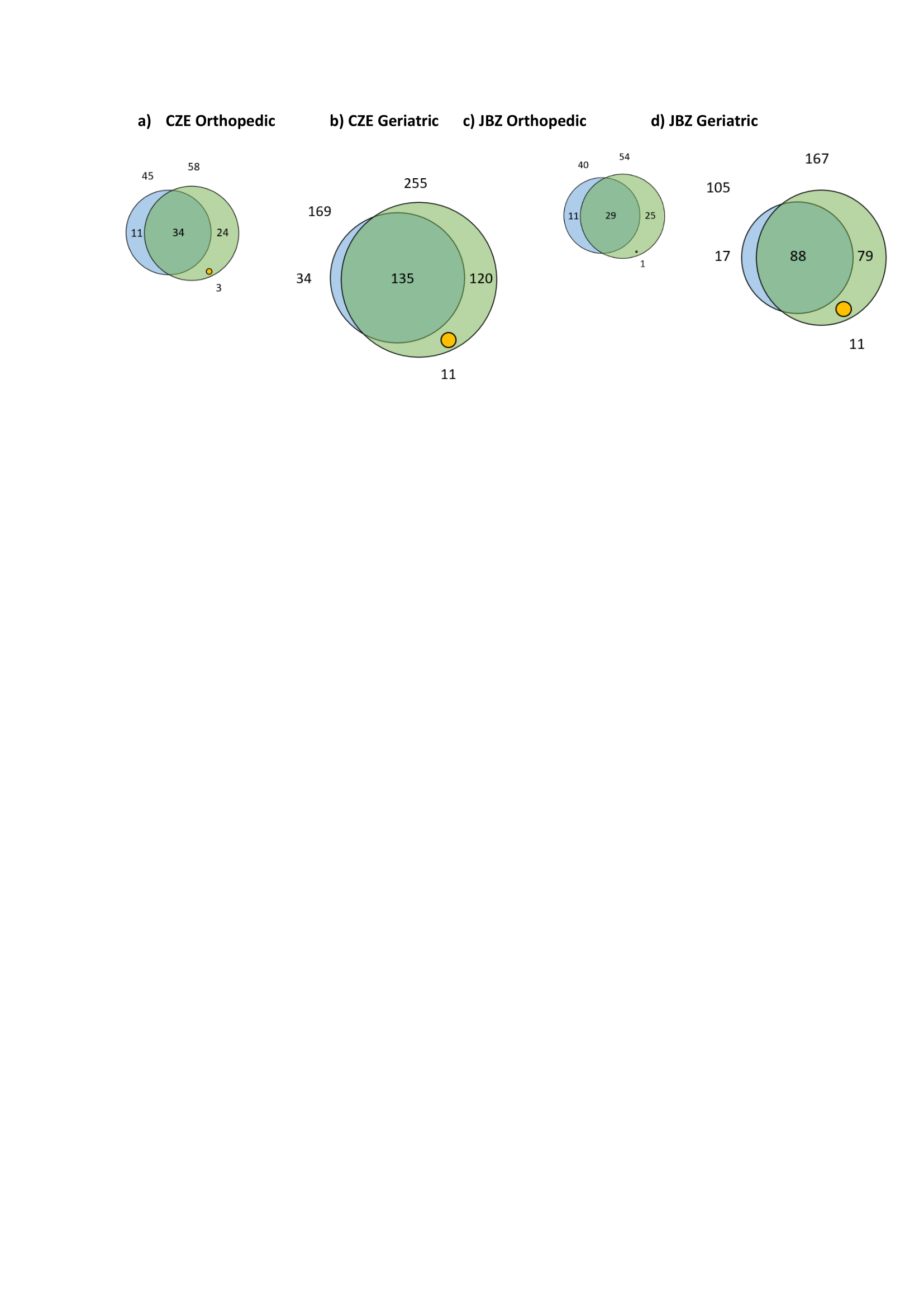

Supplement: S1 Fig — (TIFF) [file pdig.0001230.s003.tiff]
