# Supplementary material for: Portability of a text mining algorithm for detecting adverse drug reactions in electronic health records across diverse patient groups in two Dutch hospitals
Source: PLOS Digit Health. 2026 Feb 10;5(2):e0001230. doi: 10.1371/journal.pdig.0001230 (PMC12890107; doi:10.1371/journal.pdig.0001230)
Supplement: Attachment — Submitted filename: Response to Reviewers.docx [file pdig.0001230.s006.docx]

**Journal Requirements:**

1. Please ensure that your Ethics Statement is available in its entirety at the beginning of your Methods section, under a subheading 'Ethics Statement'. It must include:

We have relocated the Ethics Statement to the beginning of our method section and have added the 3 points below in the manuscript.

1) The name(s) of the Institutional Review Board(s) or Ethics Committee(s)

2) The approval number(s), or a statement that approval was granted by the named board(s)

3) (for human participants/donors) - A statement that formal consent was obtained (must state whether verbal/written) OR the reason consent was not obtained (e.g. anonymity).

2. Please upload separate figure files in .tif or .eps format. Also, remove the figures from your manuscript file but keep the legends.

For more information about figure files please see our guidelines:

https://journals.plos.org/digitalhealth/s/figures 
https://journals.plos.org/digitalhealth/s/figures#loc-file-requirements

3. Please provide an Author Summary. This should appear in your manuscript between the Abstract (if applicable) and the Introduction, and should be 150–200 words long. The aim should be to make your findings accessible to a wide audience that includes both scientists and non-scientists. Sample summaries can be found on our website under Submission Guidelines:

<https://journals.plos.org/digitalhealth/s/submission-guidelines#loc-parts-of-a-submission>

We have added an author summary between the abstract and introduction. The added text in the manuscript will read as follows:

Adverse Drug Reactions (ADRs) present a significant challenge in healthcare, with many being recorded as unstructured free-text in Electronic Health Records (EHRs). This study evaluates the portability of a text mining (TM) algorithm developed for ADR identification in EHRs, by assessing its performance across two departments in two Dutch hospitals. EHR free-text data from 62 patients in the geriatric and orthopedic departments were analyzed using manual review and the TM algorithm. The results showed that the TM algorithm demonstrated a good performance, with an F-score of 0.64, sensitivity of 80%, and positive predictive value (PPV) of 54%. Additionally, the algorithm identified clinically relevant ADRs that were missed in manual review. These findings suggest that the TM algorithm is portable across different clinical settings without requiring adaptation, highlighting its potential for broader implementation in ADR detection.

4. We have noticed that you have uploaded Supporting Information files, but you have not included a list of legends. Please add a full list of legends for your Supporting Information files after the references list.

We have added the list of legends to the supporting information files.

5. We notice that your supplementary figures and tables are included in the manuscript file. Please remove them and upload them with the file type 'Supporting Information'. Please ensure that each Supporting Information file has a legend listed in the manuscript after the references list.

We have uploaded the files with thy type “supporting information” and have added a legend list in the manuscript after the references list.

6. We note that your Data Availability Statement is currently as follows: The data underlying this article are available in the article and in its online supplementary material.
 
 Please confirm at this time whether or not your submission contains all raw data required to replicate the results of your study. Authors must share the “minimal data set” for their submission. PLOS defines the minimal data set to consist of the data required to replicate all study findings reported in the article, as well as related metadata and methods (https://journals.plos.org/plosone/s/data-availability#loc-minimal-data-set-definition).
 
 For example, authors should submit the following data:
 
 - The values behind the means, standard deviations and other measures reported;
 - The values used to build graphs;
 - The points extracted from images for analysis.
 
 Authors do not need to submit their entire data set if only a portion of the data was used in the reported study.
 
 If your submission does not contain these data, please either upload them as Supporting Information files or deposit them to a stable, public repository and provide us with the relevant URLs, DOIs, or accession numbers. For a list of recommended repositories, please see https://journals.plos.org/plosone/s/recommended-repositories.
 
 If there are ethical or legal restrictions on sharing a de-identified data set, please explain them in detail (e.g., data contain potentially sensitive information, data are owned by a third-party organization, etc.) and who has imposed them (e.g., an ethics committee). Please also provide contact information for a data access committee, ethics committee, or other institutional body to which data requests may be sent. If data are owned by a third party, please indicate how others may request data access.

Supporting file of raw data added

- Supplement - Patient and EHR characteristics
- Supplement- raw data pARDs, assessors, naranjo, serious, EHR review and recorded as structured (FILE)

7. Some material included in your submission may be copyrighted. According to PLOS’s copyright policy, authors who use figures or other material (e.g., graphics, clipart, maps) from another author or copyright holder must demonstrate or obtain permission to publish this material under the Creative Commons Attribution 4.0 International (CC BY 4.0) License used by PLOS journals. Please closely review the details of PLOS’s copyright requirements here: PLOS Licenses and Copyright. If you need to request permissions from a copyright holder, you may use PLOS's Copyright Content Permission form.

Please respond directly to this email or email the journal office and provide any known details concerning your material's license terms and permissions required for reuse, even if you have not yet obtained copyright permissions or are unsure of your material's copyright compatibility.

Potential Copyright Issues:

a. We do not publish any copyright or trademark symbols that usually accompany proprietary names, eg (R), (C), or TM (e.g. next to drug or reagent names). Therefore please remove all instances of trademark/copyright symbols throughout the text, including ‘Gaston Pharma®’ and ‘Research Manager®’.

We have removed all trademarks.

If the reviewer comments include a recommendation to cite specific previously published works, please review and evaluate these publications to determine whether they are relevant and should be cited. There is no requirement to cite these works unless the editor has indicated otherwise.

**Additional Editor Comments (if provided):**

[Note: HTML markup is below. Please do not edit.]

**Response to the reviewers’ comments:**

**Reviewers' Comments:**

Reviewer's Responses to Questions

**Comments to the Author**

1. Does this manuscript meet PLOS Digital Health’s [publication criteria](https://track.editorialmanager.com/CL0/https:%2F%2Fjournals.plos.org%2Fdigitalhealth%2Fs%2Fjournal-information%23loc-criteria-for-publication/1/010f019a90dfa0e0-582f1f14-9fca-4cc8-ba82-035f4d143886-000000/oZsvh1xknwzy8_dLyhoNsloEybYHYaio29cPVx2Coj8=237)? Is the manuscript technically sound, and do the data support the conclusions? The manuscript must describe methodologically and ethically rigorous research with conclusions that are appropriately drawn based on the data presented.

Reviewer #1: Yes

Reviewer #2: Yes

Reviewer #3: Yes

2. Has the statistical analysis been performed appropriately and rigorously?

Reviewer #1: Yes

Reviewer #2: Yes

Reviewer #3: N/A

3. Have the authors made all data underlying the findings in their manuscript fully available (please refer to the Data Availability Statement at the start of the manuscript PDF file)?

The [PLOS Data policy](https://track.editorialmanager.com/CL0/https:%2F%2Fjournals.plos.org%2Fdigitalhealth%2Fs%2Fdata-availability/1/010f019a90dfa0e0-582f1f14-9fca-4cc8-ba82-035f4d143886-000000/C1zAInD09hYW3kGn_znda-MBTbZCQVNAmsSiMjEHeWo=237) requires authors to make all data underlying the findings described in their manuscript fully available without restriction, with rare exception. The data should be provided as part of the manuscript or its supporting information, or deposited to a public repository. For example, in addition to summary statistics, the data points behind means, medians and variance measures should be available. If there are restrictions on publicly sharing data—e.g. participant privacy or use of data from a third party—those must be specified.

Reviewer #1: Yes

Reviewer #2: Yes

Reviewer #3: No

4. Is the manuscript presented in an intelligible fashion and written in standard English?

PLOS Digital Health does not copyedit accepted manuscripts, so the language in submitted articles must be clear, correct, and unambiguous. Any typographical or grammatical errors should be corrected at revision, so please note any specific errors here.

Reviewer #1: Yes

Reviewer #2: Yes

Reviewer #3: Yes

5. Review Comments to the Author

Please use the space provided to explain your answers to the questions above. You may also include additional comments for the author, including concerns about dual publication, research ethics, or publication ethics. (Please upload your review as an attachment if it exceeds 20,000 characters)

**Reviewer #1:**

Comment 1.

Statistical comparison reporting of the differences in F-score could be clearer and more standardized. Specifically, reporting of the one-tailed p-value of 0.034 suggesting potentially significant departmental difference after writing that the two-tailed test was not significant (p=0.068) adds a degree of ambiguity. This suggests the initial decision to use the two-tailed test might not cover the entire hypothesis of the authors or directional interpretation must be more convincingly justified.

We appreciate your suggestion to clarify the statistical comparison and the reporting of the p-values. After careful consideration, we agree that presenting both the two-tailed and one-tailed p-values without clear justification may create ambiguity.

The reason we initially included the one-tailed p-value (0.034) is that we had a specific hypothesis about the direction of the difference between the departments. We expected the Geriatrics department to have a higher F-score than the Orthopedic department, based on the assumption that Geriatrics patients have more complex medication regimens, which may lead to a higher incidence of ADRs being documented.

Given this directional hypothesis, we initially chose to report the one-tailed p-value (0.034) to assess whether the Geriatrics department performed significantly better in terms of F-score. However, we acknowledge that presenting both the two-tailed (p = 0.068) and one-tailed p-values without proper context may create confusion.

To address this issue and make the statistical reporting clearer, we will revise the manuscript as follows:

A) We will justify the use of the one-tailed test by explicitly stating that the hypothesis was based on the assumption that the Geriatrics department, with its more complex patient population, would document more ADRs, leading to higher sensitivity and PPV.
B) We will focus on the two-tailed p-value (0.068) as the primary result, reporting that it is not statistically significant at an alpha level of 0.05. We will also report the one-tailed p-value (0.034), but only after clarifying that it was based on our directional hypothesis.
C) We will emphasize the confidence interval (95% CI: -0.75 to 20.17), which includes zero, indicating that there is no strong evidence of a significant difference between the two departments.

The revised text in the manuscript will read as follows:

Methods: (**added**)

“**To test whether the non-surgical department demonstrated a significantly higher F-score than the surgical department, a one-tailed t-test was conducted, based on the hypothesis that non-surgical department, with its more complex patient population, would document more ADRs, leading to higher sensitivity and PPV. A two-tailed test was also performed for completeness, and results were reported accordingly.** ”

Results: (**major changes**)

The difference in F-score between the orthopedic department (M = 72.3; SD = 19.2) and the geriatric department (M = 62.6; SD =15.3) was not statistically significant (t= 1.84; p = 0.068). The 95%-confidence interval includes zero (-0.75 to 20.17), ~~which further suggests~~ **suggesting** that the **result was not statistically significant** in a two-tailed test. **However, based on the hypothesis that the Geriatrics department would document more ADRs due to the complexity of the patient population, a one-tailed p-value (0.034) suggests a potentially significant difference in the expected direction.**

Discussion: (**minor changes**)

“Although the overall F-score differences **of the two-tailed t-test** were not statistically significant, the one-tailed test **may** suggest~~s~~ a potentially meaningful variation, warranting further investigation in larger cohorts.”

We believe this revision will provide a clearer and more standardized presentation of the statistical results.

Comment 2.

Be systematic in the application of two-tailed tests, as the sample size is small and exploratory one-tailed tests are misleading in the absence of a well-defined a-priori hypothesis.

Thank you for your follow-up question. We believe the issue of statistical clarity has already been addressed in our response to the first question. Specifically, we have provided a clear justification for the use of a one-tailed test, explaining that the hypothesis was based on the expectation that the Geriatrics department would document more ADRs due to the complexity of its patient population, which would result in higher sensitivity and PPV. We hope this additional clarification sufficiently answers your concern about the consistency and justification of the statistical approach.

Comment 3.

It is suggested for the authors to mention on the importance of AI/ML integration in healthcare/IoT security, which represents a subject of data management and security—a core issue with EHR systems.

- The recommended paper: "Personal Data Protection Model in IOMT-Blockchain on Secured Bit-Count Transmutation Data Encryption Approach".
- This paper in the Background subsection after Text mining (TM), a method of NLP, has been found to be useful in deriving clinically relevant information from unstructured text sources such as nursing reports.

Thank you for your suggestion regarding the integration of AI/ML in healthcare and IoT security, specifically in relation to EHR systems. We agree that this is a critical and emerging area of research, particularly with the increasing reliance on data-driven technologies in healthcare environments.

The revised text in the manuscript will read as follows:

Background: (**added**)

Text mining (TM), a natural language processing (NLP) approach, has shown potential in extracting clinically relevant information from unstructured text sources such as nursing reports. **Furthermore, the integration of AI/ML with IoT security, highlights the critical role of advanced technologies in ensuring data protection and privacy in healthcare systems, including Electronic Health Records (EHR).** Despite its potential, the portability of TM algorithms across different settings has yielded mixed results.

Comment 4.

Employ the same term pADR (potential Adverse Drug Reaction) for mentioning the algorithm output, and ADR for the concept or those ascertained by manual verification, especially in the titles of the Results section. For example, in Figure 2 legend, blue and green circles are referred to as "ADRs" where the study refers to them as "unique pADRs" in the text.

We agree that consistency in terminology is important for clarity. In response to your comment, we will revise the manuscript to use the term "pADR" (potential Adverse Drug Reaction) for referring to the algorithm's output, and reserve the term "ADR" for referring to the concept or those ascertained by manual review. This distinction will be applied consistently throughout the manuscript, including in the Results section and figure legends. For example, in Figure 2, The blue circle including the green portion, represents the total number of unique ADRs identified by the manual electronic health record (EHR) review. The green circle represents the total number of unique pADRs identified by the TM algorithm.

Comment 5.

A) The authors cite the disputed validity of Naranjo's algorithm. Adding a brief statement that it was still used because it is one of the best accepted and simple-to-use methods would provide more explanation.

We acknowledge this important point that further clarification on the use of Naranjo’s algorithm is necessary. In response, we will add a statement in the manuscript to explain that **Naranjo** remains one of the most widely accepted and simple-to-use methods, which makes it a preferred choice in studies like ours.

The revised text in the manuscript will read as follows: (**added**)

Despite these limitations, Naranjo was the preferred method in this study **due to its wide acceptance and simplicity, making it a practical tool for causality assessment in clinical research**.

B) Statistically correct the reportage in the Results and Discussion to express clearly the lack of significant difference in the F-score between departments based on the two-tailed T-test.

See the revised text suggested in comment 1 of reviewer 1.

Comment 6.

In mentioning Text Mining (TM), incorporate literature references to the cross-pollination of Machine Learning/AI with other technologies within healthcare. This will lay the ground for subsequent discussion on the usage of combining the TM algorithm with Clinical Decision Support Systems (CDSS).

We appreciate your suggestion to enhance our discussion with AI combined with other technologies within healthcare. Therefore, we added a new Alinea to our discussion.

The added text in the discussion will read as follows:

**Artificial intelligence (AI) such as TM are being integrated with various technologies to transform healthcare. It is combined with EHRs, genomic profiles, and wearable health technologies to analyze complex datasets, predict disease progression, and recommend optimized treatment strategies. For example, in medical imaging, the integration of AI into Picture Archiving and Communication Systems (PACS) has proven crucial, increasing diagnostic accuracy by up to 93.2% and reducing diagnostic times for critical conditions such as intracranial hemorrhages by up to 90%.**

**Reviewer #2:** The authors performed important work on portability/generalizability of general ADR retrieval from natural text.

Introduction:
Comment 7.

First paragraph
The last two sentences of the first paragraph are overlapping it feels the authors are trying to convey the same thing twice., consider revision
The last sentence: HER instead of HER

Thank you for your attention to detail. We have amended the text in line with all your suggestions.

Comment 8.
Second paragraph
The authors refer to mixed results of portability, it would be helpful to have some reference of the mixed results, and were these also on the topic of ADRs.

Thank you for your comment. We agree that providing references clarifies the statement regarding mixed results of NLP system portability. As noted in the Introduction, prior studies have reported varying outcomes: for example, Mehrabi et al. (2015) [20] and Martinez et al. (2014) [21] found challenges in porting NLP systems across institutions, whereas Banerjee et al. (2023) [24] and Fabacher et al. (2023) [26] reported more successful multi-institutional implementation. Additional studies have evaluated ease of adoption and reproducibility across clinical settings, highlighting variable results depending on context and system design (Zheng et al., 2015 [22]; Johnson et al., 2018 [23]; Adekkanattu et al., 2019 [25]; Carroll et al., 2012 [27]).

These references support the assertion that portability outcomes are mixed, reflecting differences in clinical domains, dataset characteristics, and NLP system implementation. Unfortunately, to our knowledge no studies are found to have tested the portability of ADRs.

Comment 9.
Third paragraph
The sentence of although sentence seems contradiction to itself. Please revise

Thank you for pointing this out. We agree that the original phrasing could be interpreted as contradictory. We have revised the text to clarify the limitation and rationale for evaluating portability.

The revised text in the manuscript will read as follows:

“ While the algorithm was validated within a single hospital and limited to non-surgical departments, its performance in other departments and clinical settings has not yet been assessed, primarily due to potential variations in documentation policies, clinical practices, and patient demographics. “

Comment 10.
Fourth paragraph
Why did the authors choose to validate in a second hospital with the same EHR system? Also a hospital within the same region?
Now the question remains how the algorithm would perform in another Dutch region or different EHR structure.

Thank you for your comment. The decision to validate the algorithm in a second hospital using the same EHR system and within the same region was driven by the need to assess its performance in a setting with similar documentation practices and patient demographics. This was an important first step to ensure the algorithm’s reliability and applicability across different departments within the same institution, as variations in clinical practices and documentation policies could impact its performance.

However, we recognize that validating the algorithm in hospitals with different EHR systems or in other regions within the Netherlands is crucial for evaluating its broader portability and generalizability. This is a limitation of the current study, and we agree that future work should focus on assessing the algorithm’s performance in hospitals using different EHR systems and in regions with potentially different patient populations and documentation practices. (Off the record: we are exploring multiple hospitals in the Netherlands to realize this future work)

In the limitations we addressed this limitation (first paragraph of limitations) and emphasize the need for further validation in diverse healthcare settings to better understand how the algorithm performs across diverse healthcare settings with different EHRs.

Comment 11.
Major: the measure/metrics of portability or the definition have not been defined in de the aim. When is it considered portable? What decline is considered acceptable? Please elaborate on this using references.

Thank you for your feedback. We agree that adding a measurement to define when the algorithm is portable or not is necessary. Therefore, we have added this measurement to the definition of portability in the analysis of materials and methods. We also added references to elaborate the measurement.

Portability was defined as the agreement between the two methods (manual review and the TM algorithm) across both hospitals (JBZ and CZE). **The F1-score, as described by Hripcsak & Rothschild (2005), with a threshold of 0.6, was used to assess this agreement, with portability considered acceptable if the F1-score is above this value, as demonstrated in previous studies.**

Comment 12.

Methods
First paragraph
It is unclear from first read in which hospital the algorithm was devolved, also on what type of population.
We appreciate your suggestion to clarify the hospital and population details in our manuscript. We have revised the text to provide more explicit information regarding the development of the algorithm and the type of population used. Below is the updated text:

“ The performance of the previously **in CZE** developed and described TM algorithm **on non-surgical patients** for identifying pADRs was assessed in two teaching hospitals (Catharina Hospital Eindhoven [CZE] and Jeroen Bosch Hospital 's-Hertogenbosch [JBZ], the Netherlands) using EHRs of **two departments a surgical (orthopedics) and a non-surgical (geriatrics)** in both hospitals and compared to manual review of the EHRs. “

Comment 13.

And related why was this comparison chosen? Both general teaching hospitals.

See comment 10.

Comment 14.
Second paragraph
Consider moving Hospital and EHR specifics to first paragraph and merging the topics about inclusion from paragraph three.

Thank you for your thoughtful suggestion. After careful consideration, we have decided to retain the current structure of the manuscript, keeping the hospital and EHR specifics in the second paragraph. We believe that this approach enhances the clarity of the study. Moving the hospital and EHR details to the first paragraph, as suggested, might cause some confusion. Therefore, we feel it is clearer to maintain the original structure. We hope this explanation justifies our decision, and we appreciate your understanding.

Comment 15.

Fourth paragraph
It is confusing to read that referral letters for example would not be text based, please elaborate.
Also, are referral letters not digitally send by general practitioner in the Netherlands?

Thank you for your insightful comment. We apologize for the confusion. To clarify, while referral letters may indeed be in text format, most of the time these letters are scanned as PDFs and then uploaded into the Electronic Health Record (EHR) system. This means that while the content is text-based, it is often in an unstructured form (i.e., scanned images or text within PDFs), which can complicate the process of text mining and Natural Language Processing (NLP) because the system may not easily extract or interpret the text.

Regarding your second question, yes, referral letters can be sent digitally by general practitioners in the Netherlands, typically through secure e-mail systems such as Zorgmail. However, as mentioned, even when digital, these letters are often sent as PDF scans rather than as fully structured or machine-readable text, which poses challenges for NLP algorithms designed to extract information from free-text documents.

The revised text in the manuscript will read as follows:

“To assess the extent of ADRs captured in structured data, the ADR and complications modules were also reviewed. Scanned documents, such as referral letters **(often PDF scans, rather than structured text)**, were excluded due to limitations in processing non-text-based information.”

Comment 16.
Identification of pADRs
Please elaborate a bit more about the in-house developed algorithm, and, or place a clear reference here to further in the text.

Thank you for your comment. We understand the need for additional clarification regarding the in-house developed algorithm. We have expanded the explanation of the algorithm and provided a clear reference to earlier sections of the manuscript for better context. The references to Wasylewicz et al. (14), Van de Burgt et al. (37), are displayed more clearly and concisely, to provide further context and detail. Below is the updated text:

“The **earlier developed** algorithm was coded in R (Version 4.5.0, The R Foundation, Auckland, New-Zeeland (38)) and has been described in detail **in our previous studies**.(14,36~~,38~~) In brief, the algorithm was initially coded in Gaston Pharma (Gaston Medical, Eindhoven, The Netherlands), a rule-based clinical decision support system, **using 5 key strategies to identify EHR notes containing possible ADRs.** ~~and~~ ~~first~~ **This was** tested on 45 patients in the study by Wasylewicz et al.(14) In the second study by Van de Burgt et al. (37), the algorithm was ~~rewritten~~ **recoded and improved** in R, **using the same population to be able to identify and categorize pADRs.** **Hereafter** **MedDRA and SNOMED-CT were added to identify possible ADRs. The last step contained adding R-script to the algorithm to present unique ADRs**. ~~In general, the same approach to identify the pADRs as the reviewers was programmed in the TM algorithm, for example the earlier mentioned Dutch G-standard.~~”

Comment 17.

Textmining algorithm
It's unclear or there's an unclear reference to how the algorithm works and what it does. The reference is only to how the algorithm was initially designed and earlier that is used G-standard and MedDRA datasets. Please elaborate on this.

See comment 16

Comment 18.

Analysis
Major: if the aim is to assess portability of the algorithm between institutions why is it defined as the agreement between manual and TM algorithm? The sentence is very confusing to read. Isn’t the comparison of the performance between institutions and department types the real metric for portability?
Please elaborate who and did the concordance matching take place?

We understand your concern about the clarity of the definition of portability and agree that further explanation is needed regarding the analysis and the metrics used.

To clarify, portability in our study was defined as the agreement between the two methods (manual review and the TM algorithm) across different hospitals (JBZ and CZE). Initially, we needed to compare the performance of the TM algorithm with the manual review to establish baseline metrics such as sensitivity and positive predictive value (PPV). These metrics were then used to calculate the F-score, which was subsequently compared between the two hospitals and departments to assess the algorithm’s portability. Therefore, the F-score, calculated from the sensitivity and PPV, was the performance metric used to evaluate how well the TM algorithm performed across different settings.

The concordance matching, including the comparison of the manual review and the TM algorithm, was carried out by two independent reviewers who manually assessed the EHRs, while the algorithm processed the same data. This methodology ensured a robust performance comparison between the methods.

We will revise the manuscript to more clearly explain the process and focus on the comparison of performance between the two hospitals and departments. See the revised text below:

“~~The~~ portability~~,~~ was defined as the agreement between the two methods (manual review and the TM algorithm) ~~was assessed in~~ **across** both hospitals (JBZ and CZE)~~,~~ **The standard performance metric, the F-score, was used to assess this agreement.** ~~using the standard performance metric, the F-score.~~ Statistical comparisons of the agreement between the manual review and the TM algorithm’s results were conducted using an unpaired T-test on the F-score. All statistical analyses were performed using IBM SPSS Statistics, version 28 (IBM Corp., Armonk, NY).”

Comment 19

Results
First paragraph
Please elaborate on inclusion days, moreover consider moving this part to methods section

We agree that further elaboration on the inclusion criteria (specifically the "inclusion days") would improve clarity. Regarding your suggestion to move this information to the Methods section, we agree that it would be more appropriate in that section. We will revise the manuscript accordingly, ensuring that the inclusion criteria and any relevant details regarding "inclusion days" are clearly described in the ethic statement section for better clarity and flow. This is conform the journal requirements, stated by the editor.

The added text in the Ethics statement will read as follows:

**“Treating physicians identified eligible patients, who were then approached and provided with detailed study information. On inclusion days, all patients within the orthopedic department were informed about the study. Written informed consent was obtained from all participants.”**

The revised text in the Results section will read as follows:

“In total 62 patients were included, whereof 32 of the JBZ (17 for the orthopedic department, 15 for the geriatric department) and 30 of the CZE (15 for both the orthopedic and the geriatric department). ~~On inclusion days, all patients within the orthopedic department were informed about the study, which~~ **In the orthopedic department, inclusion led** ~~subsequently led to the inclusion of~~ **to** two additional patients at JBZ who expressed their willingness to participate. This resulted in 25,684 free-text EHR notes for review.”

Comment 20
Second paragraph
Second sentence seems to end abruptly, please revise

Thank you for pointing this out, we have revised the sentence.

Comment 21
The comparison between CZE and JBZ in length of stay ( median) is remarkable as the amount of notes and words is even higher in JBZ, please check if this is really correct. If this correct please elaborate on this in the discussion section also on the difference in notes.

Thank you for your insightful comment. We carefully rechecked the data and ensure the correct interpretation of the comparison between CZE and JBZ, specifically regarding the length of stay and the volume of notes. Between the two hospitals, no significant differences were observed in patient or data characteristics. Only, the length of hospitalization was significantly longer in CZE compared to JBZ. This is pointed out in the discussion section. The difference of length of stay is likely attributable to variations in the clinical indications for admission and the times of admissions in the included year. At CZE, a specialized hospital for cardiac surgery and endocarditis, two patients experienced prolonged hospitalization due to endocarditis, a condition that typically requiring prolonged inpatient care. These two patients were not statistical outliers. Several other patients in CZE also had longer hospital stays due to multiple admissions. As a result, the overall length of stay in CZE was higher than in JBZ.

Comment 22
Portability of the algorithm in two different hospitals
First sentence, please revise due to style

Thank you for pointing this out, we have revised the sentence.

**“The F-score, sensitivity, and PPV for both departments are presented in Table 2, while Figure 2 shows the Venn diagrams illustrating the overlap between the EHR review methods.”**

Comment 23

Discussion
Please elaborate on how the results ‘” highlight challenges related to variability in
documentation practices and data characteristics” as results seem very comparable.

Thank you for your comment. We appreciate your observation regarding the comparability of the results. Upon reflection, while the overall results (such as F-scores, sensitivity, and PPV) appear comparable between the two hospitals, there are still subtle but important challenges related to **variability in documentation practices** and **data characteristics** that can affect the generalizability of the algorithm. Even more, the overall results (such as F-scores, sensitivity, and PPV) were different between the two departments. Additionally, the clinical characteristics of the patient populations in different departments (e.g., orthopedic vs. geriatric) and the institutional practices at two different hospitals (CZE and JBZ) may lead to different patterns in data collection and documentation. While the performance metrics across the two hospitals and two departments are comparable, these subtle differences highlight the need for the algorithm to be adaptable to a variety of documentation styles and patient characteristics.

To further clarify this point, we have revised the discussion section as follows:

“This study provides valuable insights into the portability of a TM algorithm for identifying pADRs in EHRs. By assessing the algorithm’s performance across two hospitals and two different departments, we explored its ability to function effectively in varied clinical settings with no adaptations. **While our results demonstrate promising sensitivity and precision, variability in documentation practices and data characteristics between institutions highlighted challenges.** ~~Our results demonstrate promising sensitivity and precision, but also highlight challenges related to variability in documentation practices and data characteristics. “~~

Comment 24

Major: please elaborate on the 20% missed ADRs by the TM algorithm. What further steps need to be taken to improve sensitivity even further? This also comes back to how the algorithm works and which techniques can be equipped or mixed to improve outcome.

Thank you for your comment. We appreciate the opportunity to provide further clarification regarding the 20% missed ADRs by the TM algorithm. The 20% of ADRs that were missed by the algorithm primarily stemmed from clinical language patterns. We have discussed this in two ways in the discussion: Firstly, our findings indicate that our sensitivity and specificity are comparable to existing approaches, see the systematic review by Modi et al. in the discussion. Secondly, it is important to acknowledge the broader issue of ADR underreporting by healthcare professionals. (47) While TM algorithms for detecting ADRs in free-text can enhance identification, they cannot fully address this challenge.

However, we understand that more clarity is necessary to point this out. Therefore, we have adjusted multiple sentences in the discussion.

**Reviewer #3:**

In this work, the authors present valuable research exploring the portability of a text-mining algorithm designed to detect potential adverse drug reactions (pADRs) in free-text clinical notes. Evaluations were undertaken in two hospitals and two clinical specialties, with the algorithm remaining unmodified throughout these assessments. The identification of ADRs was performed by manual review and the algorithm. The study revealed that performance was comparable across sites and departments. Furthermore, the tool successfully identified clinical ADRs that had been overlooked during manual review. The authors conclude that the algorithm is portable across institutions using the same EHR platform.

Thank you for your positive and thoughtful feedback. We appreciate your recognition of the value of our research exploring the portability of the text-mining algorithm for detecting potential adverse drug reactions in free-text clinical notes.

The results provide interesting findings related to the question if NLP algorithm for ADR detection can be used across hospitals. However, there are some opportunities for improvement:

Comment 25
- Even though testing across two hospitals makes the work more generalizable than single-site studies, only 62 patients were included. While the inclusion of over 25,000 free-text EHR notes provides a rich data source for analysis, the small number of patients per subgroup limits generalizability. The manuscript would improve by including an explanation why this sample size is sufficient or expanding the cohort.

Thank you for the constructive comment. We recognize that the sample size of 62 patients, while inclusive of data from two hospitals, might raise concerns regarding generalizability, particularly with regard to subgroups.

In our study, we aimed to balance practical feasibility and the richness of the data, especially given the large 25,000+ free-text EHR notes included in the analysis. The decision to limit our sample size was guided by the need to achieve sufficient power to detect meaningful patterns, while being mindful of logistical constraints. However, we fully acknowledge the importance of addressing the limitations posed by the relatively small patient sample size.

Regarding the statistical power, we believe that our current sample size is sufficient for the type of analysis we conducted, especially using prior data (fist paper: 340 ADRs, 45 patients, mean of 7,55 and standard deviation of 6,59, correct registered ADRs 2%, difference in population 7,35). The calculation done in “power and sample size program” suggested that a cohort of 18 subjects in the control group and 18 subjects in the experimental group would provide sufficient power (90%) to detect meaningful differences in the algorithm's performance, with a Type I error rate of 5%. This suggests that even with the relatively small cohort in each subgroup, we can reasonably detect meaningful differences where they exist, assuming a similar effect size.

That said, we agree that expanding the cohort would strengthen the study and enhance its generalizability. Therefore, we expanded the inclusion to 30 patients per hospital.

Incorporating these points, we have revised the manuscript to include a more detailed explanation of why we believe this sample size is sufficient, based on statistical considerations, while acknowledging the potential benefits of a larger cohort for future studies. We hope this explanation addresses your concerns and helps clarify the rationale behind our design choices.

The revised text in the manuscript will read as follows:

“Inclusion criteria were patients hospitalized during November 2022 until December 2023 for a minimum of 24 hours in the geriatrics or orthopedics departments at either hospital. **To ensure adequate power for statistical analysis,** each department was required to include a minimum of 15 patients, resulting in a total of at least 60 participants across both hospitals. **The sample size was determined based on a power calculation, utilizing data from two previously conducted studies. This calculation indicated that a cohort of this size would provide sufficient statistical power (0.9) to detect meaningful differences, with a Type I error rate of 0.05.”**

Comment 26
- For manual review an agreement of 65% is reported but reasons for discrepancies are unclear. Providing a formal agreement measure and a brief explanation of disagreement sources would strengthen confidence in the manual review as the study’s reference standard.

Thank you for your valuable feedback. We have carefully considered your comments regarding the need for a formal agreement measure and the unclear reasons for discrepancies. In response, we have included the Cohen’s Kappa of 0.58 as a formal measure of inter-assessor agreement, which clarifies the reliability of our manual review process. Additionally, we have provided an explanation of the sources of discrepancies, which were resolved through consensus or with the help of a third assessor. The third assessor was not explained in the results, even though we described this in our method section. Your comment also led us to identify errors in our data that had not been noticed before. Thanks to your observation, we were able to correct these issues, further enhancing the accuracy of our data. We believe these revisions in the text and figure 1 strengthen the robustness of our study and improve confidence in our reference standard for ADR assessment.

The adjusted text in the manuscript:

“A total of 747 ADRs were identified by the two assessors. During matching, 58 potential reADRs were detected and **234** ADRs were identified as duplicates. **Excluding the duplicates and reADRs resulted in 455 unique ADRs. 61% (n=278) of the included ADRs was identified by both assessors, 18% (n=81) was identified by one of both assessors and included after consensus discussion between both assessors and/or third assessor. 96 of the pADRs identified by one of the assessors were not included. This resulted in 359 unique ARDs.** No ADRs with a Naranjo score < 1 were found, which resulted in 359 unique included ADRs remained. **The assessors had a Cohen’s Kappa of 0.58 of the included ADRs, indicating moderate agreement. The discrepancies in ADR identification included variances in how clinical terminology was interpreted and the subjective nature of certain notes, which were resolved through consensus or by a third assessor.**”

Comment 27
- The results rely primarily on p-values without consistent reporting of confidence intervals. Including 95% CIs for key comparisons would enhance transparency and help readers assess the precision and practical significance of the findings.

Thank you for your valuable feedback regarding the reliance on p-values in our analysis. We agree that the inclusion of 95%-CI would enhance the transparency of the results, allowing readers to better understand the range of possible values for the population parameter, rather than just whether a result is statistically significant. To address this concern, we have now added 95% confidence intervals for the key comparisons in the revised manuscript in the result section.

Comment 28
- A PPV of 54% means half of the flagged pADRs were incorrect, but this is not analyzed. The inclusion of examples of common false positives and discussing ways to reduce them would be interesting.

Thank you for your comment regarding the Positive Predictive Value (PPV) of 54% and the importance of addressing false positives. We agree that exploring and analyzing the sources of false positives would strengthen the manuscript, especially as this would provide further context for the flagged pADRs and potential strategies to improve detection accuracy. In response to your comment, we have added the top three false positives in the discussion and how we think we can prevent them in the revised manuscript.

Top 3:

1. Incorrect association of the medication with the ADR:

Examples: CVA for which thrombolysis with actilyse was performed; confusion regarding solifenacin intake; risk of falling in epileptics, consider start fentanyl for pain

In this case, the flagged pADR was incorrectly linked to the medication, despite it being used for a different clinical indication. The algorithm could have falsely associated medications with unrelated conditions or used imprecise terms. We recommend incorporating a step in the algorithm to exclude medications based on specific indications or use stricter rule-based systems to connect medications with precise clinical contexts to improve the accuracy.

2. Replacement of one drug by another for medical reasons:

Example: Rivaroxaban to 15 mg once daily due to loss in renal function

This is a case of a medical substitution for a clinical reason rather than an adverse reaction. To reduce such false positives, we suggest adding a more nuanced rule for medication changes, ensuring the system recognizes these as legitimate clinical decisions rather than ADRs.

3. Patient refusal of medication due to potential side effects:

Example: Patient refuses alendronate due to concern about esophageal cancer risk

This case flagged as a potential ADR due to the patient’s refusal to take the medication because of perceived risks. However, this is not an actual adverse event but rather a patient concern or preference. We recommend developing a filtering mechanism to differentiate between patient-reported concerns about medications and actual adverse events, which would help reduce such false positives.

Revised discussion section in the manuscript:

**“False positives where mainly due to three reasons: 1) Incorrect association of the medication with the ADR; 2) Replacement of one drug by another for medical reasons; 3) Patient refusal of medication due to potential side effects. The improvements we would suggest are: incorporating a step to exclude medications based on specific indications, adding a more nuanced rule for medication changes, ensuring the system recognizes these as legitimate clinical decisions and developing a filtering mechanism to differentiate between patient-reported concerns about medications and actual ADRs.”**

6. PLOS authors have the option to publish the peer review history of their article ([what does this mean?](https://track.editorialmanager.com/CL0/https:%2F%2Fjournals.plos.org%2Fdigitalhealth%2Fs%2Feditorial-and-peer-review-process%23loc-peer-review-history/1/010f019a90dfa0e0-582f1f14-9fca-4cc8-ba82-035f4d143886-000000/9cBylDCqN3gwX2NfE2lPk3EDvScTuoZQ-stVSXchGk4=237)). If published, this will include your full peer review and any attached files.


**Do you want your identity to be public for this peer review?** If you choose “no”, your identity will remain anonymous but your review may still be made public.


For information about this choice, including consent withdrawal, please see our [Privacy Policy](https://track.editorialmanager.com/CL0/https:%2F%2Fwww.plos.org%2Fprivacy-policy/1/010f019a90dfa0e0-582f1f14-9fca-4cc8-ba82-035f4d143886-000000/MiHuhLSInosxndH5Inu5EVAtRgbNodFfSAeX5ji4SHM=237).

Reviewer #1: No

Reviewer #2: No

Reviewer #3: No

[NOTE: If reviewer comments were submitted as an attachment file, they will be attached to this email and accessible via the submission site. Please log into your account, locate the manuscript record, and check for the action link "View Attachments". If this link does not appear, there are no attachment files.]

**Figure resubmission:**

While revising your submission, we strongly recommend that you use PLOS’s NAAS tool (https://ngplosjournals.pagemajik.ai/artanalysis) to test your figure files. NAAS can convert your figure files to the TIFF file type and meet basic requirements (such as print size, resolution), or provide you with a report on issues that do not meet our requirements and that NAAS cannot fix.

After uploading your figures to PLOS’s NAAS tool - https://ngplosjournals.pagemajik.ai/artanalysis, NAAS will process the files provided and display the results in the "Uploaded Files" section of the page as the processing is complete. If the uploaded figures meet our requirements (or NAAS is able to fix the files to meet our requirements), the figure will be marked as "fixed" above. If NAAS is unable to fix the files, a red "failed" label will appear above. When NAAS has confirmed that the figure files meet our requirements, please download the file via the download option, and include these NAAS processed figure files when submitting your revised manuscript.

**Reproducibility:**

To enhance the reproducibility of your results, we recommend that authors of applicable studies deposit laboratory protocols in protocols.io, where a protocol can be assigned its own identifier (DOI) such that it can be cited independently in the future. Additionally, PLOS ONE offers an option to publish peer-reviewed clinical study protocols. Read more information on sharing protocols at https://plos.org/protocols?utm_medium=editorial-email&utm_source=authorletters&utm_campaign=protocols
